# Evaluating an Association Between Prenatal Smoking Behavior and Exclusive Breastfeeding: A Population-Based Study

**DOI:** 10.1177/15598276231206121

**Published:** 2023-10-14

**Authors:** Dinesh Dharel, Rudra Dahal, Kamala Adhikari, Nazeem Muhajarine, Asmita Bhattarai

**Affiliations:** 1 Department of Community Health and Epidemiology, 7235 University of Saskatchewan, Saskatoon, SK, Canada (DD, NM); 2 Department of Pediatrics, 12357 University of Alberta, Edmonton, AB, Canada (DD); 3 Department of Health Sciences, 4512 University of Lethbridge, Lethbridge, AB, Canada (RD); 4 Department of Community Health Sciences, Cumming School of Medicine, 70401 University of Calgary, AB, Canada (RD, KA, AB); 5 Provincial Population and Public Health, Alberta Health Services, Calgary, AB, Canada (KA)

**Keywords:** association, behavior, prenatal smoking, exclusive breastfeeding, pregnancy outcomes

## Abstract

**Background:** Prenatal smoking is consistently associated with adverse breastfeeding outcomes. We aimed to evaluate the association of smoking cessation or continuation during pregnancy with exclusive breastfeeding in a representative sample of the general Canadian population. **Methods:** We used the pooled sample of 9860 females with pregnancy experience of last under-five child from the Canadian Community Health Surveys 2015-2018 public use microdata file. We categorized self-reported prenatal smoking status as continuing, quitting, or no smoking. We evaluated the association between exclusive breastfeeding for 6 months or more with prenatal smoking status using multivariable logistic regression, adjusted for socio-demographic variables. **Results:** With the pooled prevalence of 33.2% (95% CI 31.7, 34.8), 34.4% (95% CI 32.8, 36.1) of non-smokers, 25.7% (95% CI 20.2, 32.2) of those who quit and 15.7% (95% CI 10.8, 22.2) of those who continued smoking reported exclusive breastfeeding for 6 months or more. Continuing smoking had lower odds of exclusive breastfeeding (aOR .47; 95% CI 0.30,0.75) but quitting smoking had no difference (aOR .78;95% CI 0.56,1.08) when compared to non-smokers. **Conclusion:** Continuing smoking during pregnancy was associated with lower rates of exclusive breastfeeding of infants for 6 months or more. Smoking cessation interventions during prenatal visits may improve exclusive breastfeeding rates.

“This study focused on smoking behavior during pregnancy as primary exposure, exclusive breastfeeding as an outcome of interest and adjusted for socio-demographic factors.”

## Background

Smoking during pregnancy is a leading preventable cause of adverse perinatal outcomes and is associated with suboptimal breastfeeding behavior.^1–6^ During pregnancy, smoking decreases uteroplacental circulation, leading to lower maternal weight gain and results in adverse fetal outcomes such as small for the gestational age, low birth weight, short stature, compromised fetal neurological development, cognitive disabilities, abortion, and premature birth.^7^

Pregnancy offers a window of opportunity for smoking cessation owing to the perceived risk to infant health outcomes. Only a modest proportion of women quit smoking during pregnancy despite the reduced risk of tobacco-related harms from smoking cessation.^1^ The ideal time to quit smoking is before pregnancy, but quitting at any time is always better than continuing smoking. The risk of preterm birth was comparable among those who never smoked vs. those who quit smoking before pregnancy, but the risk was higher compared to non-smokers if they quit smoking in the second trimester.^8^ Studies have shown significant gains in pregnancy outcomes if the cessation occurs in the first trimester.^8,9^ Although adverse birth outcome remains significant despite quitting smoking later in pregnancy, there are some health benefits nonetheless.^10^

The benefits of breastfeeding for infants, lactating mothers, and society are well-established in the literature.^11–13^ Breastfeeding mothers have lower rates of medical concerns such as type 2 diabetes, hypertension, hyperlipidemia, cardiovascular diseases, ovarian cancer, and breast cancer.^14,15^ Similarly, breastfed infants are at lower risks of obesity, ear/respiratory/gastrointestinal infections, eczema, leukemia, and sudden death syndrome.^16^

Exclusive breastfeeding (EBF) is defined as “receiving breast milk (including expressed milk) and no other liquid (including water) or solid foods except nutritional supplement and medications.”^17^ Various studies found that specific socio-demographic characteristics such as age, parity, educational level, and smoking habits can impact the initiation and duration of EBF.^18–20^ In Canada, more than 90% of women initiate EBF; however, there is a sharp decline and reaches 20.1% in the first month, and the rate stands at 9.8% and 10.6% before the 6 months and 6 months, respectively.^21^ Statistics from the Canadian Maternal Experience Survey (2006-2007) indicated that 14.4% of mothers exclusively breastfed for 6 months or mom to their babies,^22^ reaching 26% in 2011-2012 and 35% in 2022.^23^ These rates are under the World Health Assembly (2012) target of 50% EBF till 6 months by 2025 and 70% by 2030.^24^

Smoking is associated with reduced milk production owing to decreased serum prolactin levels due to nicotine, a shorter duration of breastfeeding^25–27^, and early weaning compared to mothers who were not smokers.^28^ Previous research concluded that maternal smoking was the strongest predictor of not initiating breastfeeding.^16^ However, in population-based studies, the literature is limited to assessing the association between smoking during pregnancy and exclusive breastfeeding practice. So, we aimed to evaluate the association between cessation and continuation of smoking during pregnancy compared to the non-smokers with exclusive breastfeeding practice in a representative Canadian sample.

## Methods

### Data Source

We used a public use microdata file from the Canadian Community Health Surveys (CCHS), a cross-sectional survey of the Canadian population aged 12 and older in all 10 Canadian provinces and three territories, conducted annually. The survey is representative of 98% of the Canadian general population and excludes full-time members of the military, institutional residents, those in very remote areas, and First Nations Peoples who live on the reserve. The CCHS has been used in past Canadian studies on prenatal smoking,^29,30^ as has the Maternity Experiences Survey.^31^ Details about the survey regarding its methodology, questionnaires, reporting guides, response rates, and data accuracy can be found on the Statistics Canada website.^32^

We pooled 2 recent cycles (2015-2016 and 2017-2018) of CCHS, from which use public-use microdata files were available. Both cycles ask the same set of questions about prenatal smoking and breastfeeding behaviors from all eligible females (i.e., those aged 15 to 55 who gave birth within the past 5 years of the survey; pooled n = 11,024; 4.9% of the whole CCHS sample) through the Maternal experiences-MEX module. The MEX module was a core content that asked about maternal experiences during the last childbirth. The module was not administered to proxy respondents. We additionally dropped the respondents with missing values (10.5%) who did not answer questions on the exposure or outcome variables. The remaining sample included 9860 females who had maternal experience with a child under 5 years of age at the time of the survey.

### Study Variables

We used self-reported smoking status in pregnancy as the exposure variable. We defined the exposure into 3 categories based on the response to the following 2 questions: I) “In the 3 months before your pregnancy with [your last child] or before you realized you were pregnant, did you smoke cigarettes?” and II) “During the last three months of your pregnancy, did you smoke?” The reference category “non-smoker” constituted those who answered “no” to the first question. Those who answered “yes” to the second question were categorized as “continued smoking during pregnancy.” The category “quit smoking during pregnancy” constituted those females who answered “yes” to the first question but “no” to the second question. We did not use the response from the question “During last pregnancy, did you smoke daily, occasionally, or not at all?” as used in other studies using prior rounds of CCHS to define the prevalence of smoking during pregnancy as we were interested specifically in exclusive breastfeeding outcome among those who continued or quit smoking during pregnancy compared to those who did not smoke prior to pregnancy. We hypothesized that those who smoked in the last 3 months of pregnancy would have a stronger inverse association with exclusive breastfeeding outcomes.

We used the derived variable “MEXDVBM6- Exclusively breastfed for 6 months or more” as the outcome variable, defined as “yes” or “no” for exclusive breastfeeding. The category “yes” includes “women who exclusively breastfed their last baby for six months or more and who may or may not still be breastfeeding.” The “no” category included those who did not exclusively breastfeed for less than 6 months and those who had not breastfed the last child at all.^33^ Exclusivity of breastfeeding is defined as feeding only breast milk without any additional liquid (even water) or solid food for a specified period to a particular infant given birth by a woman. This definition of exclusive breastfeeding is consistent with the most reported definition of exclusive breastfeeding. The duration is based on the question, “How long did you breastfeed or give breast milk to [your last child]?” Exclusive breastfeeding duration for 6 or more months is most reported in the literature and by Health Canada to align with global and national recommendations.^11,34^

Other study variables included socio-demographic characteristics that have been shown to affect prenatal smoking and exclusive breastfeeding.^31,35,36^ These include the female’s age (20-24 years, 25-34 years, or 35 years and above), immigrant status (immigrant, non-immigrant), marital status (single, married, or common-law), education (less than high school, secondary, or post-secondary), employment status in past 12 months (employed, non-employed), and the annual household income in Canadian dollars (less than $20,000, 20,000-79,999, or 80,000 and above).

### Statistical Analysis

The prevalence of exclusive breastfeeding was similar in the CCHS 2015/2016 cycle (31.8%: 95% CI 29.7,34.1) and CCHS 2017/2018 cycle (34.5%: 95% CI 32.4,36.7), with the chi-square *P*-value of .07. Hence, the cycles were pooled, and further analyses were conducted in the pooled sample.

Descriptive statistics were presented as percentages and corresponding 95% Confidence Intervals (CIs). The distribution of sample characteristics in the overall sample and according to exclusive breastfeeding status was assessed using the chi-square test (P-value <.05).

We used univariable and multivariable logistic regression to examine the association between exclusive breastfeeding and smoking status during pregnancy. Socio-demographic variables known to be associated with exclusive breastfeeding and smoking, including maternal age, education, immigration status, household income, employment status, and marital status, were included in the regression model for adjustment. The estimates were then presented as Odds Ratios (ORs) and 95% CIs.

We also conducted a sensitivity analysis in a restricted sample where the non-smokers were excluded. We ran an adjusted logistic regression model to directly examine whether prenatal smoking status (continuing smoking in the third-trimester vs. quitting smoking after finding out about the pregnancy) is significantly associated with exclusively breastfeeding.

Sampling weights were applied when estimating proportions and Odds Ratios (ORs) to address the unequal selection probabilities inherent to the study design, according to the Statistics Canada guideline. The independent variables included had <5% missing values, and thus complete case analysis was performed. Data management and analyses were conducted using STATA software version 16.

The Public Use Microdata File is de-identified and publicly available; review and approval by the research ethics board are not required.

## Results

The distribution of sample characteristics in the overall sample and that stratified by the breastfeeding status is shown in Table 1. Most of the females in the sample were aged 35 years and above, Canadian-born, married, had post-secondary or university education, and were in the high-income group. In our sample, 4.8% of women quit smoking during pregnancy while 4.1% continued smoking in the last 3 months of pregnancy.

**Table 1.** Distribution of Sample Characteristics in the Overall Sample and Stratified by Exclusive Breastfeeding Status in Canada During 2015-2018.

Characteristics^a^	Overall% (95% CI)Unweighted n = 9860 Weighted n = 2,860,387	Exclusive Breastfeeding% (95% CI) Unweighted n = 3219 Weighted n = 950,535.91	Not exclusive Breastfeeding% (95% CI) Unweighted n = 6641 Weighted n = 1,909,851	Chi-square *P*-value
**Maternal age (unweighted** **n** **= 9860; weighted n = 2,860,387)** 20-24 years 25-34 years 35 years and above	5.5 (4.8,6.2) 55.6 (53.9,57.2) 39.0 (37.4,40.6)	3.3 (2.6,4.3) 52.4 (49.5,55.2) 44.3 (41.5,47.1)	6.5 (5.7,7.5) 57.1 (55.2,59.1) 36.3 (34.4,38.3)	<.001
**Immigration status (unweighted n = 9588; weighted n = 2,770,103)** Canadian-born Immigrant for 0-9 years Immigrant for ≥ 10 years	71.8 (70.1,73.5) 15.9 (14.6,17.4) 12.2 (11.0,13.6)	66.9 (63.9,69.9) 20.1 (17.5,23.0) 12.9 (10.9,15.3)	74.2 (72.1,76.2) 13.9 (12.3,15.6) 11.9 (10.4,13.6)	<.001
**Maternal education (unweighted n = 9799; weighted n = 2,843,157)** Post-secondary or university degree Secondary graduation Less than secondary graduation	76.2 (74.8,77.5) 17.5 (16.3,18.7) 6.4 (5.7,7.1)	79.8 (77.6,81.9) 15.2 (13.3,17.2) 5.0 (4.0,6.2)	74.3 (72.6,76.0) 18.6 (17.1,20.1) 7.1 (6.2,8.0)	<.001
**Household income (unweighted n = 9845; weighted n = 2,859,337)** $80,000 or more $20,000 to $79,999 No income or <$20,000	53.6 (51.9,55.2) 37.7936.2,39.3) 8.7 (7.8,9.7)	55.6 (52.8,58.4) 37.6 (34.8,40.4) 6.8 (5.5,8.4)	52.6 (50.6,54.5) 37.8 (35.9,39.7) 9.6 (8.5,10.9)	<.001
**Employment status (last 12 months) (unweighted n = 9819; weighted n = 2,848,057)** Employed Unemployed	24.8 (23.4,26.2) 75.2 (73.8,76.6)	28.3 (25.8,31.0) 71.7 (69.0,74.2)	23.0 (21.3,24.8) 77.0 (75.2,78.7)	<.001
**Marital status (unweighted n = 9833; weighted n = 2,857,509)** Married/common-law Single/Divorced/Widowed	86.5 (85.5,87.5) 13.5 (12.5,14.5)	91.7 (90.3,92.9) 8.3 (7.1,9.7)	84.0 (82.5,85.3) 16.0 (14.7,17.5)	<.001
**Smoking status (unweighted n = 9853; weighted n = 2,858,434)** Non-smoker prior to pregnancy Quit smoking during pregnancy Smoked in the last 3 months of pregnancy	91.1 (90.3,91.9) 4.8 (4.2,5.5) 4.1 (3.6,4.7)	94.4 (93.0,95.5) 3.7 (2.8,4.9) 1.9 (1.3,2.9)	89.5 (88.4,90.5) 5.3 (4.6,6.2) 5.2 (4.5,5.9)	<.001

^a^The proportions are calculated excluding missing values; the sample size varies by characteristics due to missing values. The proportions reported are weighted according to Statistics Canada guidelines.

The overall prevalence of exclusive breastfeeding for 6 months or more in the pooled sample (n = 9860) was 33.2% (95%CI:31.7,34.8). The prevalence of exclusive breastfeeding was higher among those who did not smoke before pregnancy (34.4%; 95%CI 32.8,36.1) followed by those who quit smoking during pregnancy (25.7%; 95%CI 20.2,32.2) and lowest among those who continued to smoke in the last 3 months of pregnancy (15.7%; 95%CI 10.8,22.2). The sample characteristics were significantly associated with exclusive breastfeeding status.

The unadjusted and adjusted estimated associations between exclusive breastfeeding status and the exposure variable (smoking status during pregnancy) and selected co-variables are shown in Table 2. When adjusted for other co-variables, the odds of exclusive breastfeeding were significantly lower among those who continued smoking in the third trimester of pregnancy compared to those who did not smoke prior to pregnancy (adjusted OR .47; 95% CI 0.30,0.75). However, the odds of exclusive breastfeeding were not significantly different for mothers who quit smoking after finding out about pregnancy compared to those who did not smoke prior to pregnancy (adjusted OR .78; 95% CI 0.56,1.08). In the sensitivity analysis conducted in a sample that excluded non-smokers (final sample size = 1172), it was found that the odds of exclusive breastfeeding were significantly lower among those who continued smoking in the third trimester as compared to those who quit smoking after finding out about pregnancy (adjusted OR .59 (95% CI 0.36,0.99) when adjusted for the socio-demographic variables.

**Table 2.** Association Between Exclusive Breastfeeding and Smoking Status During pregnancy, and Socio-demographic Covariates in Canada, 2015/2016 and 2017/2018 CCHS (Unweighted n = 9860; Weighted n = 2,860,387).

Characteristics^a^	Unadjusted Odds Ratio (95% CI)	*P*-value	Adjusted Odds Ratio (95% CI)	*P*-value
**Smoking status in pregnancy** Non-smoker prior to pregnancy Quit smoking during pregnancy Smoked in the last 3 months of pregnancy	Reference .66 (.48,0.91) .35 (.23,0.55)	.012 <.001	Reference .78 (.56,1.08) .47 (.30,0.75)	.135 .001
**Maternal age** 20-24 years 25-34 years 35 years and above	Reference 1.80 (1.32,2.46) 2.40 (1.74,3.30)	<.001 <.001	Reference 1.38 (.98,1.96) 1.75 (1.22,2.50)	.067 .002
**Immigration status** Canadian-born Immigrant for 0-9 years Immigrant for ≥ 10 years	Reference 1.61 (1.29,2.00) 1.20 (.94,1.55)	<.001 .144	Reference 1.30 (1.02,1.64) 1.03 (.80,1.34)	.032 .807
**Maternal education** Less than secondary graduation Secondary graduation Post-secondary certificate diploma or university degree	Reference 1.16 (.85,1.58) 1.52 (1.16,2.00)	.034 .002	Reference 1.13 (.82,1.56) 1.30 (.97,1.75)	.445 .080
**Household annual Income** No income or <$20,000 $20,000 to $79,999 $80,000 or more	Reference 1.41 (1.06,1.86) 1.50 (1.14,1.97)	.004 .019	Reference 1.23 (.90,1.70) 1.22 (.87,1.71)	.196 .242
**Employment status (past 12 months)** Unemployed Employed	Reference 1.32 (1.13,1.55)	.001	Reference 1.39 (1.16,1.69)	.001
**Marital status** Single/Widowed/divorced/separated Married/common-law	Reference 2.11 (1.73,2.56)	<.001	Reference 1.59 (1.26,2.00)	<.001

^a^The ORs are weighted according to Statistics Canada guidelines.

In the adjusted model, the exclusivity of breastfeeding was also positively associated with a maternal age 35 years or more, being an immigrant residing in Canada for less than 10 years, being employed in the last 12 months, and being married or living with a common-law partner.

## Discussion

This study explored the association between prenatal smoking and exclusive breastfeeding among Canadian pregnant women. Our analysis confirmed the negative relationship between continuing smoking during pregnancy with exclusive breastfeeding of infants for 6 months or more. Mothers who smoke represent a high-risk group for a shorter duration of breastfeeding.^28,37^ An Australian study concurred with our finding, showing that the continuation of smoking is the strongest determinant of suboptimal exclusive breastfeeding.^38^ However, the exclusive breastfeeding outcome was not statistically different among those who quit smoking during pregnancy compared to those who never smoked. In addition, the odds of exclusive breastfeeding were significantly lower among those who continued smoking in the third trimester as compared to those who quit smoking after finding out about the pregnancy, in sensitivity analysis. These findings suggest that discontinuation of the high-risk (smoking) behavior after finding out about pregnancy would encourage optimum breastfeeding behavior. Our findings are relevant in the context of the “desire to protect a baby” having been previously described as an important facilitator to quitting smoking during pregnancy.^39^

Smoking during pregnancy is a modifiable risk factor that is associated with adverse maternal, fetal, and neonatal outcomes.^9,40–43^ Maternal smoking habits can influence females to make the decision to breastfeed their newborn or not.^20^

The negative associations between smoking and exclusive breastfeeding had been described in literature even before the 1950s.^44^ Females who smoke cigarettes are less likely to initiate breastfeeding compared to those who are non-smokers.^45^ Multiple research findings have shown such a negative relationship and have recommended smoking prevention and cessation interventions as the promoting methods of boosting breastfeeding duration.^19,25,46^

Independent of prenatal smoking status, the exclusivity of breastfeeding for 6 months or more of age was positively associated with being advanced maternal age (35 years or more), new immigrant (residing in Canada for less than ten years), employed (in the last 12 months) and married or living with a common-law partner. These findings are consistent with previous studies that suggest a pattern of social disparities in the breastfeeding outcome in high-income countries including Canada, with relatively better breastfeeding behavior with higher maternal age, income, education, and presence of a partner. The relationship of exclusive breastfeeding rate with immigration status and duration is probably dynamic and needs further exploration.^34^ Several factors are known to play a role in exclusive breastfeeding.^47^ Those include maternal educational level,^33^ age,^48^ employment status of the mother,^49^ marital status,^50^ antenatal classes,^51^ and maternal psychological factors.^52^ Although our data did not demonstrate a statistically significant difference in the association of higher maternal education and annual household income with higher rates of exclusive breastfeeding in infants 6 months or more, the trend observed was consistent with previous studies^53,54^ that demonstrated a higher level of maternal socioeconomic status, higher annual income, and higher educational attainment being associated with better breastfeeding outcome. The reason for not observing the expected strong association is not entirely apparent but this may also suggest the equitable support families receive irrespective of socioeconomic status in Canada.

This study focused on smoking behavior during pregnancy as primary exposure, exclusive breastfeeding as an outcome of interest and adjusted for socio-demographic factors. Food insecurity has been shown to be associated with the initiation and exclusivity of breastfeeding in previous studies using data from Canada and the United States.^55,56^ However, our dataset had 16.5% missing values in the food insecurity variable and was excluded from the analysis. The effect of early initiation of breastfeeding on exclusive breastfeeding has also been studied in the Haitian context.^15^ The data on the initiation of breastfeeding in CCHS is limited to dichotomous “yes” or “no” responses to the question “Did you breastfeed or try to breastfeed your baby, even if only for a short time?” and does not measure whether the initiation of breastfeeding was within the first hour of birth or not, which precludes analysis of early initiation of breastfeeding the Canadian context. The study of the initiation of breastfeeding as an outcome variable is precluded from this study as those who did not initiate breastfeeding are a subset of a category of our outcome variable, ‘no exclusive breastfeeding. It is also important to explore whether females who smoke during pregnancy are behaviorally more likely not to breastfeed or if they produce less milk because of smoking.^16^ Future studies should also examine the impact of vaping during pregnancy on breastfeeding outcomes, potentially comparing vaping with cigarette smoking or with no smoking or vaping.

### Strength and Limitations

This study utilized data from a nationally representative survey of the Canadian household population which follows a robust methodology. We combined the 2 most recent cycles of CCHS for which a public use microdata file is available and have similar and revised survey design and methodology which allowed for analysis in the pooled sample to increase the sample size and thus enhance the power of the study. We were able to categorize the prenatal smoking behavior into those who continued and quit smoking during pregnancy and compare those 2 categories in the sensitivity analysis, in addition to a primary comparison of both categories with the non-smokers. Such a comparison of prenatal smoking behaviors in relation to the exclusivity of breastfeeding has not been previously reported.

However, the measure of the smoking status of pregnant women was self-reported and did not include biochemical validation of cigarette use. Studies have shown that self-reported measures of smoking status by pregnant women may be underestimated (Dietz et al., 2011; Hannover et al., 2008), and the smoking cessation rates in pregnancy may be overestimated (England et al., 2007). Moreover, the measurement of exposure (smoking behavior) and the outcome (exclusive breastfeeding) in surveys is prone to bias due to the social desirability to report good behavior. In addition, recall bias is possible in both exposure and outcome due to the inclusion of women with maternal experience as remote as up to 5 years prior to the survey. Given the cross-sectional nature of the CCHS, the maternal characteristics derived from other modules do not necessarily apply exactly to the period during the pregnancy with the last child from which the MEX experiences data on the exposure variable (prenatal smoking cessation or continuation) and the outcome variable (exclusive breastfeeding for 6 months or more) are derived.

Smoking behavior during pregnancy in our study was categorized based on self-reported smoking history within 3 months prior to knowing pregnancy and 3 months prior to delivery and did not consider the relapse of smoking after the delivery among the quitters. However, postpartum smoking behavior can be different from smoking behavior during pregnancy, with relapse within 1 year after childbirth in nearly two-thirds of females who quit during pregnancy.^57^ Future studies on breastfeeding outcomes should consider the effect of postpartum smoking too. In addition, the timing of smoking cessation during the pregnancy may have a variable impact on breastfeeding behavior; however, we could not assess this due to data unavailability. CCHS does not measure the severity or dose of smoking during pregnancy, which precluded a dose-response analysis of prenatal smoking behavior with the exclusivity of breastfeeding. For future research, we suggest studying the dose-response of smoking during pregnancy and the timing of cessation during the pregnancy with the early initiation and exclusivity of breastfeeding.

## Conclusion

We observed significantly lower rates of exclusive breastfeeding of infants for 6 months or more among females who continued smoking during pregnancy compared to those who did not smoke prior to knowing about pregnancy but no significant difference among those who quit smoking after finding out about pregnancy when compared to non-smokers. Therefore, smoking cessation efforts should be targeted and managed in prenatal visits to establish exclusive breastfeeding for 6 months or more.
